# Click It or Ticket Campaign — May 20–June 2, 2013

**Published:** 2013-05-17

In 2011, approximately 21,000 passenger vehicle occupants (excluding motorcyclists) died in motor vehicle crashes in the United States, representing 66% of all motor vehicle crash deaths (1). An additional 2.6 million occupants were treated for injuries in emergency departments (2). Although seat belt use in the United States reached 87% overall, millions of persons continue to travel unrestrained (3). Using a seat belt is one of the most effective means of preventing serious injury or death in the event of a crash. Seat belts saved an estimated 11,949 lives in 2011. If everyone had been buckled up, an estimated 3,400 additional lives could have been saved (4).

Click It or Ticket, a national campaign coordinated annually by the National Highway Traffic Safety Administration to increase the proper use of seat belts, will be conducted May 20–June 2, 2013. Law enforcement agencies across the nation will conduct intensive, high-visibility enforcement of seat belt laws during both daytime and nighttime hours. Nighttime enforcement of seat belt laws is encouraged because seat belt use is lower at night (1). Campaign activities in 2013 focus on the need for all adults and all children who have outgrown booster seats* to buckle up on every trip. Additional information about the 2013 Click It or Ticket campaign activities is available at http://www.nhtsa.gov/PEAK. Additional information on preventing motor-vehicle crash injuries is available at http://www.cdc.gov/motorvehiclesafety.

## Footnotes

* Guidelines for child passenger safety are available at http://www.cdc.gov/motorvehiclesafety/child_passenger_safety/cps-factsheet.html.
